# Supplementary material for: Cholesterol, Amyloid Beta, Fructose, and LPS Influence ROS and ATP Concentrations and the Phagocytic Capacity of HMC3 Human Microglia Cell Line
Source: Int J Mol Sci. 2023 Jun 20;24(12):10396. doi: 10.3390/ijms241210396 (PMC10299308; doi:10.3390/ijms241210396)
Supplement: Supplementary file 1 [file ijms-24-10396-s001.zip › ijms-2455481-supplementary.pdf]

Cholesterol, amyloid beta, fructose and LPS influence ROS and ATP concentrations and the phagocytic capacity of HMC3 hu-man microglia cell line

Oscar M. Muñoz Herrera[1], Brian V. Hong[1], Ulises Ruiz Mendiola[2], Izumi Maezawa[2], Lee-Way Jin[2], Carlito B. Lebrilla[3], Danielle J. Harvey[4], Angela M. Zivkovic[1*]

[1]Department of Nutrition, UC Davis; [2]Department of Pathology and Laboratory Medicine, UC Davis Medical Center; [3]Department of Chemistry, University of California; [4]Department of Public Health Sciences, UC Davis; Davis, California 95616 United States

* Correspondence: amzivkovic@ucdavis.edu; Tel.: +1-530-752-3973

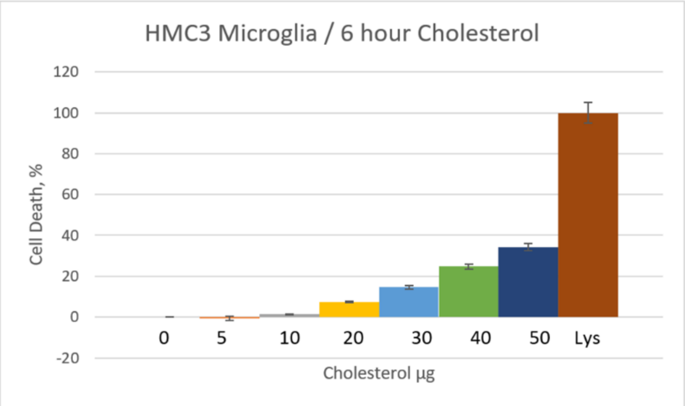

Supplemental Figure S1. HMC3 Cytotoxicity Assay. Cholesterol loaded HMC3 microglia (varying doses), for 6 hours.

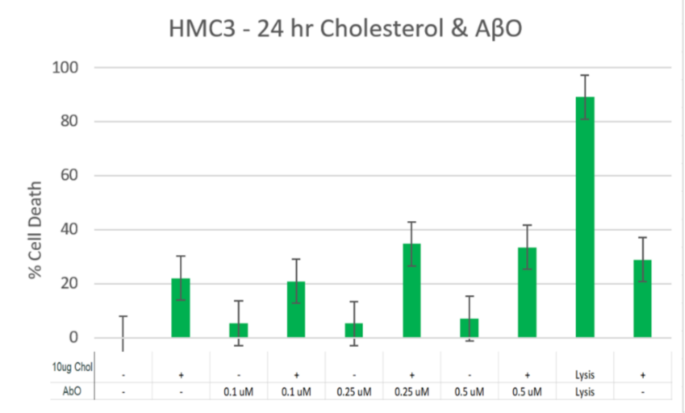

Supplemental Figure S2. HMC3 Cytotoxicity Assay. Cholesterol (10µg/mL) loaded and AβO treated HMC3 microglia, for 24 hours.

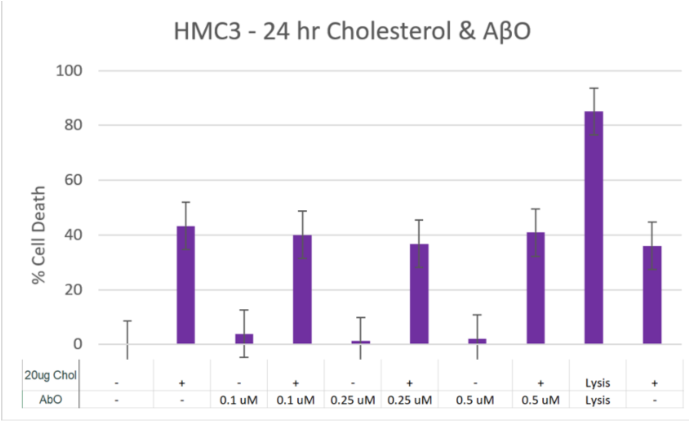

Supplemental Figure S3. HMC3 Cytotoxicity Assay. Cholesterol (20μg/mL) loaded and AβO treated HMC3 microglia, for 24 hours.

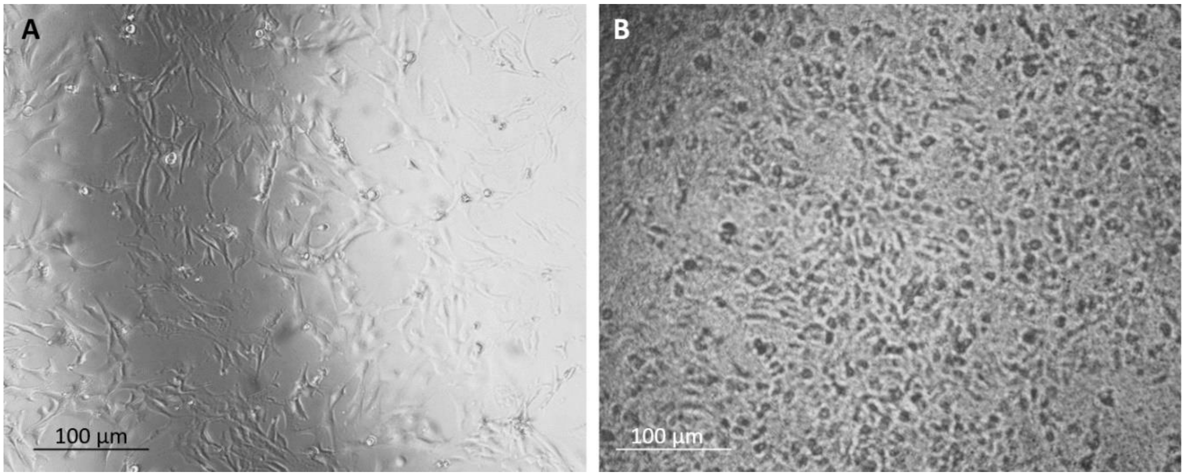

Supplemental Figure S4. Effects of treatments on HMC3 microglia morphology A) Untreated HMC3 microglia at timepoint 0 hours, on 96-well plates. n=3, three biological replicates. Digital Microscope Camera was used for the picture. 10x magnification, scale bar 100 μm. B) HMC3 microglia following the 24-hour period treatment with AβO+Fru+Chol+LPS. n=3, three biological replicates. Digital Microscope Camera was used for the picture. Terms: Lipopolysaccharide (LPS), amyloid beta oligomer (AβO), cholesterol (Chol), fructose (Fru). 10x magnification, scale bar 100 μm.

Supplemental Figures S5. Effects of treatments on HMC3 microglia morphology, continued.

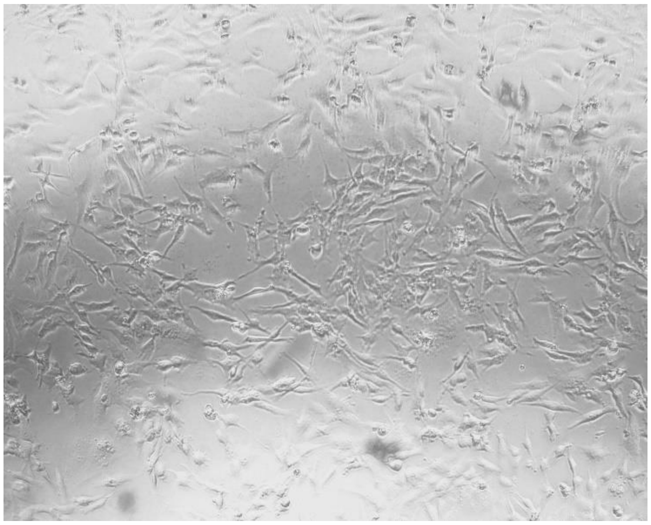

A) HMC3 microglia, at 24 hour time-point, following amyloid-beta oligomer (AβO) treatment. 10x magnification.

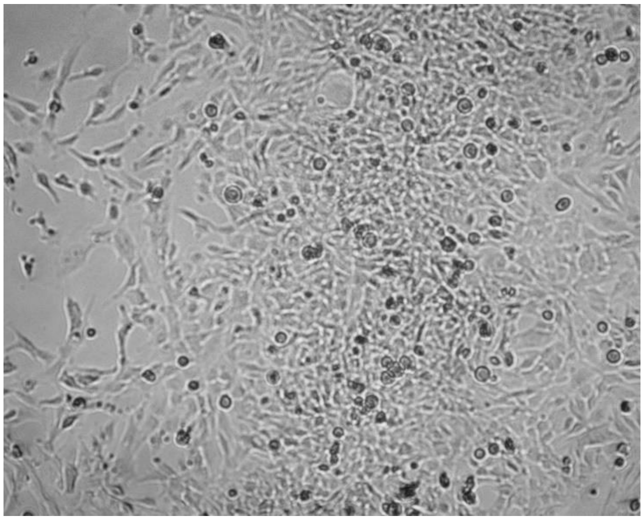

B) HMC3 microglia, at 24 hour time-point, following fructose (Fru) treatment. 10x magnification.

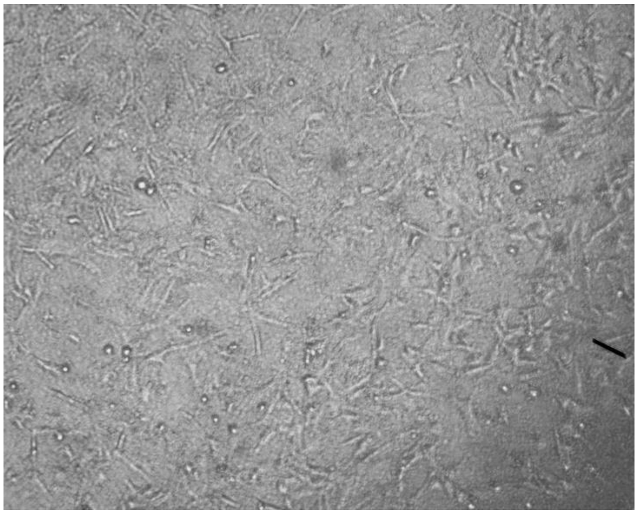

C) HMC3 microglia, at 24 hour time-point, following cholesterol (Chol) treatment. 10x magnification.

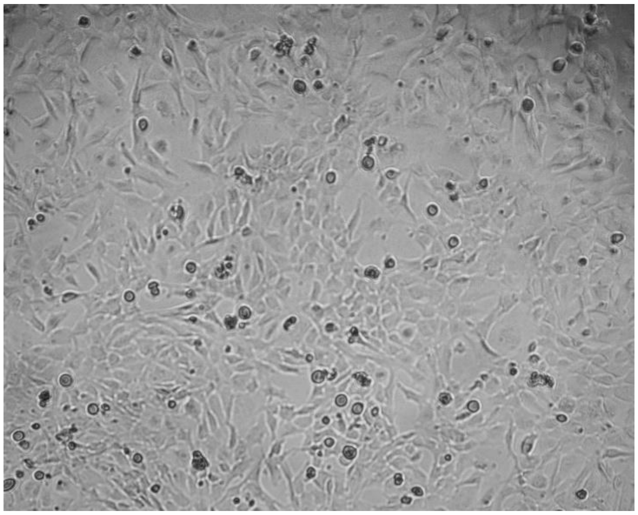

D) HMC3 microglia, at 24 hour time-point, following Lipopolysaccharide (LPS) treatment. 10x magnification.

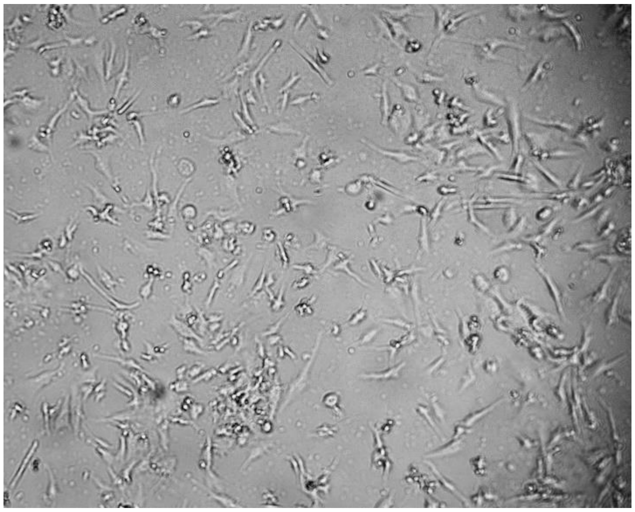

E) HMC3 microglia, at 24 hour time-point, following AβO+Fru treatment. 10x magnification.

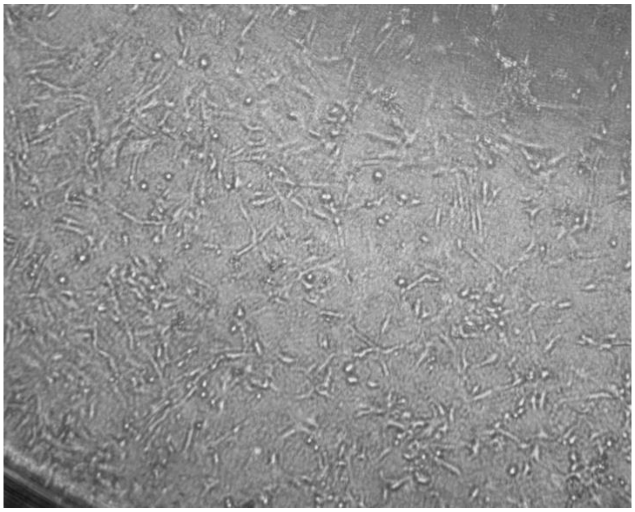
F) HMC3 microglia, at 24 hour time-point, following AβO+Chol treatment. 10x magnification.

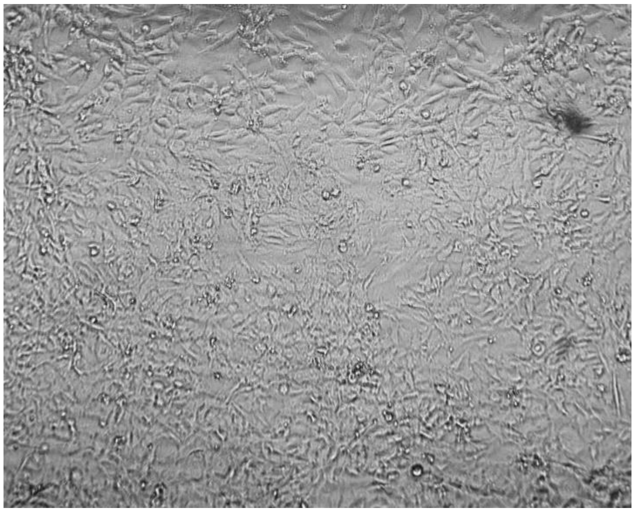
G) HMC3 microglia, at 24 hour time-point, following AβO+LPS treatment. 10x magnification.

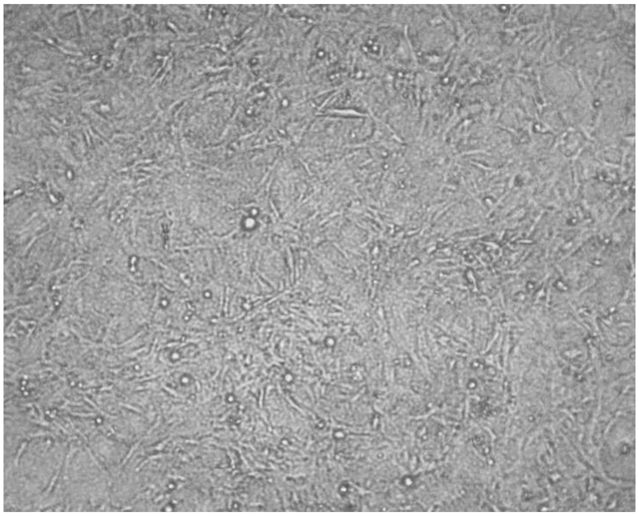
H) HMC3 microglia, at 24 hour time-point, following Fru+Chol treatment. 10x magnification.

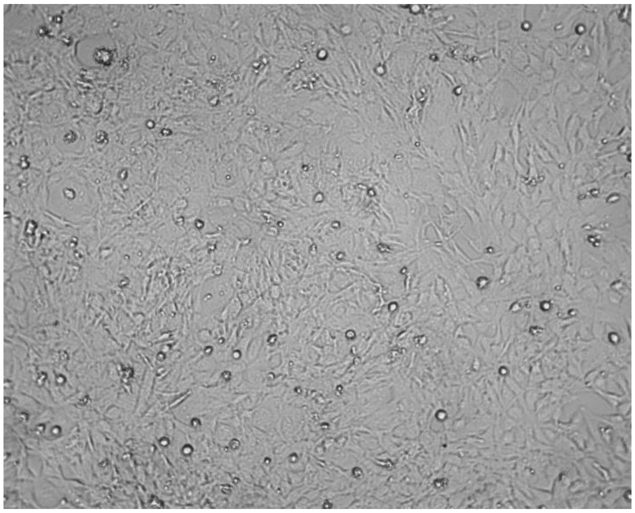
I) HMC3 microglia, at 24 hour time-point, following Fru+LPS treatment. 10x magnification.

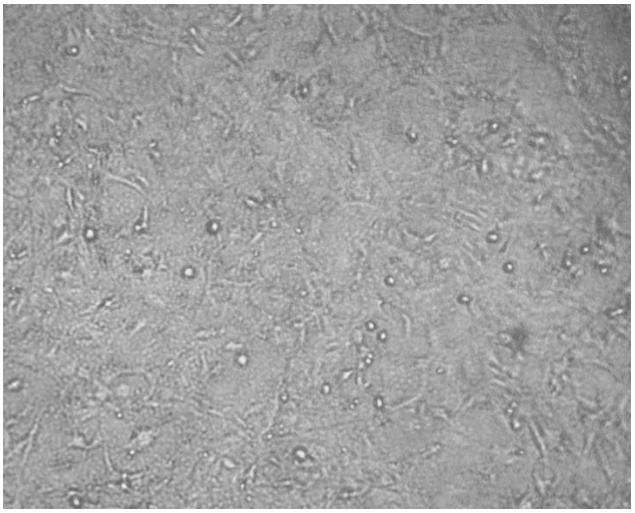
J) HMC3 microglia, at 24 hour time-point, following Chol+LPS treatment. 10x magnification.

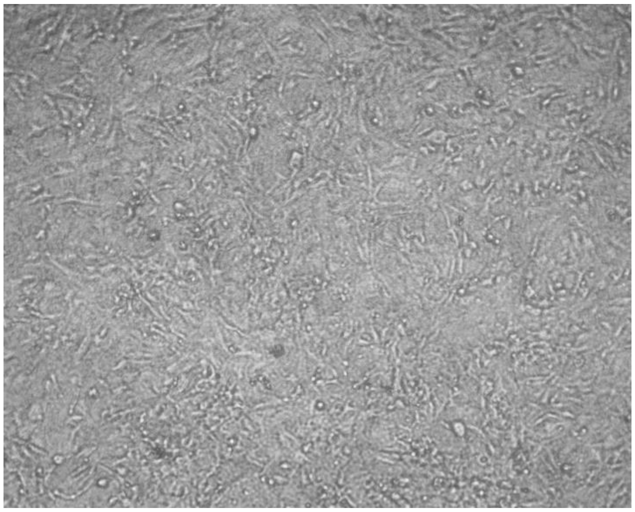
K) HMC3 microglia, at 24 hour time-point, following AβO+Chol+LPS treatment. 10x magnification.

Table S1. Log2 Fold change of treatment on expression of pro-inflammatory cytokine genes and APOE in HMC3 microglia compared to control.

Treatment	IL1B	IL6	TNFA	APOE
AβO	-1.76	-0.27	-0.59	0.52
Cholesterol	-2.40	0.01	1.00	-1.43
Fructose	-0.72	-0.11	-0.54	0.94
LPS	2.97	3.93	3.08	0.54
AβO_Cholesterol	-3.19	-1.29	0.62	1.39
AβO_Fructose	-1.55	0.09	0.63	1.68
AβO_LPS	1.49	3.61	3.39	1.46
Cholesterol_LPS	1.56	5.07	3.57	0.96
Fructose_Cholesterol	-1.30	0.61	0.94	1.57
Fructose_LPS	2.90	3.90	2.61	0.36
AβO_Cholesterol_LPS	-2.87	0.87	1.76	2.51
AβO_Fru_Chol_LPS	-1.78	1.46	4.14	3.32

Table S2. Mean ± SD of the effects of treatments on whole-cell total and esterified cholesterol concentrations, mitochondrial total cholesterol concentrations and ApoE secretion in HMC3 microglia.

	Total Cholesterol		Esterified Cholesterol		Mitochondrial Cholesterol		ApoE	
Treatment	Mean (μM)	SD	Mean (μM)	SD	Mean (μM)	SD	Mean (pg/mL)	SD
Control	5.27	1.78	1.79	0.06	7.73	0.33	638.28	110.96
AβO	8.21	0.02	3.81	0.19	9.16	0.02	299.93	38.96
Cholesterol	8.05	0.26	3.14	0.24	10.45	0.25	541.90	81.65
Fructose	12.20	1.22	5.79	1.25	10.44	2.04	587.79	59.47
LPS	6.81	0.48	3.48	0.42	10.51	1.05	575.70	53.48
AβO_Cholesterol	8.70	0.18	3.36	0.04	11.50	2.98	341.23	40.86
AβO_Fructose	10.14	0.26	5.76	0.34	10.10	0.41	330.39	158.83
AβO_LPS	8.55	0.03	3.87	0.21	9.14	0.32	348.32	93.38
Cholesterol_LPS	9.33	1.68	3.67	0.53	11.08	1.00	503.94	66.78
Fructose_Cholesterol	12.45	2.01	4.34	0.11	10.20	0.29	441.36	70.09
Fructose_LPS	10.76	0.50	5.60	0.81	10.93	0.28	478.91	54.36
AβO_Cholesterol_LPS	11.90	0.05	5.24	1.06	11.04	0.40	365.01	48.45
AβO_Fru_Chol_LPS	11.04	0.26	6.42	0.27	14.10	3.36	326.21	80.64

Table S3. Mean ± SD of the effects of treatments on HMC3 microglia ROS and ATP concentrations and phagocytic activity.

	ATP		ROS		Phagocytosis	
Treatment	Mean (nmol/well)	SD	Mean (Log2Fluorescence)	SD	Mean (Log2Fluorescence)	SD
Control	1.93	0.43	12.77	0.04	12.98	0.62
AβO	1.72	0.21	13.52	0.50	10.64	0.24
Cholesterol	1.66	0.21	13.35	0.46	11.89	0.20
Fructose	1.92	0.22	12.23	0.47	11.97	0.13
LPS	2.01	0.24	12.37	0.61	11.68	0.37
AβO_Cholesterol	1.54	0.26	13.42	0.44	11.89	0.08
AβO_Fructose	1.60	0.17	13.30	0.22	9.10	0.71
AβO_LPS	1.70	0.31	13.26	0.26	9.95	0.11
Cholesterol_LPS	1.80	0.29	13.05	0.33	13.14	0.37
Fructose_Cholesterol	1.68	0.17	12.21	0.46	12.31	0.60
Fructose_LPS	1.87	0.24	12.30	0.43	11.51	0.80
AβO_Cholesterol_LPS	1.53	0.22	13.07	0.24	11.97	0.59
AβO_Fru_Chol_LPS	1.27	0.13	15.51	0.45	10.73	0.67
